# Lipotoxicity, lipid peroxidation and ferroptosis: a dilemma in cancer therapy

**DOI:** 10.1007/s10565-025-10025-7

**Published:** 2025-04-26

**Authors:** Chuhan Ma, Huixin Hu, Hao Liu, Chongli Zhong, Baokang Wu, Chao Lv, Yu Tian

**Affiliations:** Department of General Surgery, Shengjing Hospital of China Medical University, Shenyang, 110004 Liaoning Province China

**Keywords:** Lipid peroxidation, Ferroptosis, Active aldehyde, Oxidized lipid, Immune cells

## Abstract

The vulnerability of tumor cells to lipid peroxidation, driven by redox imbalance and lipid overabundance within the tumor microenvironment (TME), has become a focal point for novel antitumor strategies. Ferroptosis, a form of regulated cell death predicated on lipid peroxidation, is emerging as a promising approach. Beyond their role in directly eliminating tumor cells, lipid peroxidation and its products, such as 4-hydroxynonenal (HNE), exert an additional influence by damaging DNA and shaping an environment conducive to tumor growth and metastasis. This process polarizes macrophages towards a pro-inflammatory phenotype, dampens the antigen-presenting capacity of dendritic cells (DCs), and undermines the cytotoxic functions of T and NK cells. Furthermore, it transforms neutrophils into pro-tumorigenic polymorphonuclear myeloid-derived suppressor cells (PMN-MDSCs). The lipid peroxidation of stroma cells also contributes to tumor progression. Although advanced nanotherapies have shown the ability to target tumor cells precisely, they often overlook the nuanced effects of lipid peroxidation products. In this review, we highlight a synergistic mechanism in which lipid peroxidation products and ferroptosis contribute to an immunosuppressive state that is temporally distinct from cell death. This insight broadens our understanding of ferroptosis-derived immunosuppression, encompassing all types of immune cells within the TME. This review aims to catalyze further research in this underexplored area, emphasizing the potential of lipid peroxidation products to hinder the clinical translation of ferroptosis-based therapies.

**Graphical abstract:**

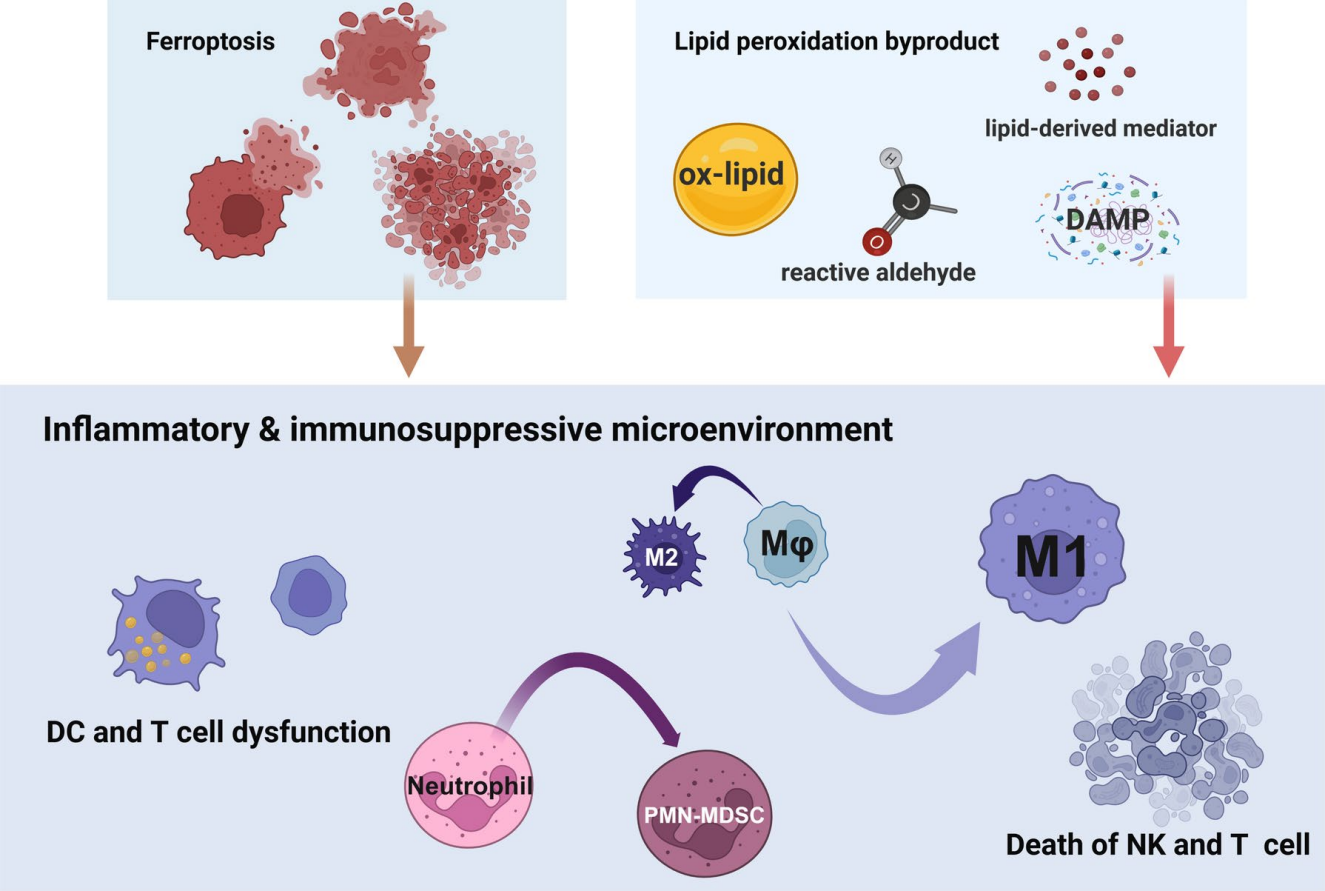

## Highlights

**• **Lipid peroxidation with anti-tumor roles shapes an inflammatory and immunosuppressive tumor microenvironment to facilitate tumor progression.

**• **Lipid peroxidation products contribute to the separation of ferroptosis-mediated immunosuppression and actual cell death in time.

**• **Long-term lipid peroxidation treatment, such as ferroptosis induction, may be detrimental to patient prognosis owing to lipid peroxidation products.

## Introduction

Reactive Oxygen Species (ROS) are a group of molecules or ions with high oxidative activity. Radicals are a major component of ROS that can attack lipids, especially polyunsaturated fatty acids (PUFAs), in a process called lipid peroxidation (Box 1). Lipid peroxidation can be divided into enzyme- and non-enzyme-dependent reactions. The enzyme-dependent pathway involves the conversion of lipids into specific mediators, such as prostaglandin E2 (PGE2), via enzymes, including cyclooxygenase (COX) and lipoxygenase (LOX). The role of these enzyme-mediated processes in cancer is well understood. Therefore, this review emphasizes the non-enzymatic processes in which ROS directly interact with lipids. Typically, it results in the destruction of lipid bilayers and the production of reactive aldehydes, both detrimental to cell survival.

Lipid accumulation and oxidative stress increase the susceptibility of tumor cells to lipid peroxidation (Clemente et al. 2020), making lipid peroxidation induction a promising anti-tumor strategy. Ferroptosis, an iron-dependent cell death that is essentially a type of lipid peroxidation reaction, has gained widespread attention in cancer research in recent years (Box 1) (Fernández-Acosta et al. 2025; Lei et al. 2024). The experiments conducted by Fan, R. et al. have demonstrated that Oxaliplatin-Artesunate (OART), as a novel ferroptosis inducer, exhibits considerable bioactivity both in vitro and in vivo, and have validated its feasibility in tumor immunotherapy (Fan 2024). Experiments by Freitas, F. P. et al. and Li, Z. et al. have elucidated the roles of 7-dehydrocholesterol reductase (DHCR7) and lysophosphatidylcholine acyltransferase 1 (LPCAT1) in fostering tumor growth. These enzymes have been shown to confer ferroptosis resistance to cancer cells, thereby enhancing their survival (Freitas 2024; Li 2024). Collectively, these findings reinforce the notion that ferroptosis holds significant cytotoxic potential against cancer. However, this process also affects immune cells within the tumor microenvironment (TME), potentially fostering an inflammatory environment that could hinder anti-tumor immunity and aid tumor development (Kim et al. 2023; Tang et al. 2023). Therefore, accurately targeting tumor cells while sparing immune cells in the TME is crucial for the application of ferroptosis in cancer therapy. Recent advancements in nanotherapies have begun to address this challenge (Clemente et al. 2020)For instance, the highly acidic intra-organelle compartment of cancer cells can quickly convert zero-valent iron nanoparticles (ZVI-NPs) into iron ions, which contributes to the precise induction of ferroptosis in tumor cells (Hsieh 2021). However, most studies have not thoroughly considered the role of lipid peroxidation products such as HNE and oxidized phospholipids (Astudillo et al. 2023; Liang et al. 2022). In our opinion, these products play a similar role to ferroptosis, possibly leading to an underestimation of their impact in previous studies. This review summarizes the effects of intracellular lipid peroxidation and its products on both tumor and immune cells, linking ferroptosis to lipid peroxidation products. This connection opens new avenues for research and highlights the potential challenges in the clinical application of ferroptosis induction, offering a fresh perspective on this complex topic.

## Methodology

Data for this review were identified by searches of PubMed (pubmed.ncbi.nlm.nih.gov) and references from relevant articles using the search terms: (“Immune cells” or “T cell” or “Macrophage” or “Neutrophil” or “MDSC” or “Dendritic cell” or “NK cell” or “Endothelial cell” or “Fibroblast” or “Adipocyte”) and (“Lipid peroxidation” or “Ferroptosis”). Only articles published in English up to the Mar 15, 2025 were included. We endeavored to comprehensively incorporate relevant studies. However, the literature selection process may inevitably be subject to selection bias influenced by the authors'subjective judgments.

## The effect of lipid peroxidation on tumor cells

Lipid metabolic reprogramming is a hallmark of malignancies. To adapt to hypoxic and nutrient-deficient microenvironments, tumor cells upregulate enzymes for de novo fatty acid synthesis primarily through steroid regulatory element-binding proteins (SREBPs). This process predominantly generates monounsaturated fatty acids (MUFA) (Snaebjornsson et al. 2020). Concurrently, tumor cells enhance PUFA uptake via transport molecules like CD36, which is influenced by the lipid composition available in the TME (Snaebjornsson et al. 2020). PUFAs are more prone to peroxidation than MUFAs. Tumor cells with redox imbalance can reduce PUFA incorporation into phospholipids as a defense mechanism against ROS (Dierge 2021; Zeng 2025). However, the increased total amount of PUFA increases their susceptibility to lipid peroxidation. Even minor disturbances in the TME can trigger substantial intracellular peroxidation (Jin 2023; Jin et al. 2023). Ferroptosis, a cell death that is dependent on lipid peroxidation, is a particularly significant anti-tumor strategy (Box 2). Tumor cells have heightened sensitivity to ferroptosis, further exacerbated by oncogene activation, hypoxia inducible factor (T) expression, and epithelial-to-mesenchymal transition (EMT) (Friedmann Angeli et al. 2019), which also positions lipid peroxidation, especially ferroptosis, as a compelling target for anti-tumor strategies.

Lipid peroxidation leads to the formation of several reactive products such as HNE, malondialdehyde (MDA), acrolein (ACR), and 4-hydroxy-hexenal (HHE). These compounds can react with cellular proteins to form Schiff bases or Michael adducts via a process known as lipoxidation (Box 1). By covalently modifying functional proteins (e.g., metabolic enzymes, receptors) in a cell type-dependent manner, lipid peroxidation-derived adducts destabilize protein structures or interfere with molecular interactions. This process ultimately enables these reactive lipid species to elicit distinct pathological responses in tumor cells or immune cells (Martín-Sierra et al. 2019; Viedma-Poyatos et al. 2021).The propensity for lipoxidation depends on the abundance, reactivity, as well as accessibility of the nucleophilic sites of proteins (Martin-Sierra et al. 2019), which varies based on the unique protein and membrane lipid compositions and detoxification enzyme expression in different tumor cells or immune cells (Dalleau et al. 2013).

HNE, one of the most reactive lipid peroxidation products, is involved in tumor cell proliferation and differentiation (Gasparovic et al. 2017). Low levels of HNE are often associated with increased proliferative activity in tumor cells (Dianzani 1993; Huang 2025). HNE inhibits the proliferation of MDA-MB- 231 cell line by modifying peptidylprolyl cis/trans-isomerase A1 (Pin1), a key regulator of the cell cycle (Aluise 2012). Furthermore, HNE can lead to the inactivation of membrane-associated catalase in tumor cells, resulting in increased lipid peroxidation in membrane plasma and apoptosis induced by hydroxyl radicals (Jakovčević 2020). Increased HNE concentrations near tumor tissues may augment resistance against tumor invasion into non-malignant areas, a response attributed to localized inflammatory reactions (Barrera 2015; Jaganjac and Zarkovic 2022).

While HNE exhibits anticancer properties, its therapeutic potential is constrained by high reactivity, poor solubility, and chemical instability. To address these limitations, researchers developed β-cyclodextrin-polyacryloylmorpholine (PACM-βCD) nanocapsules, which stabilize HNE and enhance its antiproliferative, pro-apoptotic, and differentiation-inducing effects across tumor cell lines, with minimal toxicity to normal cells (Pizzimenti 2015). Topical administration in 3D skin melanoma models demonstrates therapeutic promise (Pizzimenti 2013; Pizzimenti 2015). Notably, inhibition of RLIP76-mediated GS-HNE transport to elevate intracellular HNE levels achieved sustained complete remission in melanoma, kidney, colon, and lung cancer xenografts in nude mice (Singhal et al. 2006; Singhal et al. 2007; Singhal et al. 2009).

However, beyond its protective effects, HNE is implicated in DNA damage and the promotion of tumorigenesis. HNE can lead to the inactivation of the tumor suppressor gene Live kinase B1 (LKB1) (Wagner et al. 2006). In addition, HNE promotes immunosuppression within the TME. It induces COX2 expression and PGE2 production in Caco- 2 cells, contributing to an environment conducive to tumor progression (Tammali et al. 2006). Aldose reductase inhibition blocks HNE-induced upregulation of COX2 and PGE2, thereby curbing tumor growth in nude mice (Tammali et al. 2006). HNE is also associated with the expression of transforming growth factor β (TGF-β), which is known for its immunosuppressive effects, as well as its involvement in the cell cycle arrest and apoptosis of cancer cells (Zanetti 2003). The intricate interplay of HNE with various cellular processes underscores its dualistic nature in tumor development and progression.

In the progression of colorectal cancer (CRC), a paradox exists in which lipid peroxidation levels decrease with the downregulation of TGF-β1 receptors in the TME (Biasi 2002). This phenomenon may be linked to the adaptive responses of tumor cells as CRC advances. Intriguingly, colon cells with adenomatous polyposis coli (APC) gene mutations express high levels of biotransformation enzymes that metabolize HNE (Baradat 2011). CRC cells also upregulate arylacetamide deacetylase (AADAC) to prevent intracellular lipid peroxidation to facilitate liver colonization (Sun 2022). Similarly, hepatocellular carcinoma (HCC) cells, when compared to normal hepatocytes, demonstrate distinct metabolic behaviors, including increased expression of metabolic enzymes and reduced intracellular HNE (Canuto et al. 1993; Zhong 2017), highlighting the adaptation of tumor cells to lipid peroxidation. A key player in this process is the nuclear factor erythroid 2-related factor 2(Nrf2)—Kelch-like ECH-associated protein 1 (Keap1) pathway, which regulates the antioxidant defense mechanisms of tumor cells (Torrente and DeNicola 2022). HNE disrupts this balance by inhibiting Keap1, leading to upregulation of Nrf2 (Levonen 2004; Numazawa et al. 2003), (Huang et al. 2012). This upregulation confers a degree of resistance to radiotherapy and chemotherapeutics, which rely on generating oxidative stress in tumor cells (Torrente and DeNicola 2022). Therefore, the levels of HNE vary depending on the specific tumor microenvironment. The dual functionality of HNE, exhibiting both pro-tumor and anti-tumor effects, is influenced by the histological origin of the tumor, its stage, and the metabolic state of the host (Martín-Sierra et al. 2019).

Oxidized lipids also act as signaling molecules that influence tumor cell phenotypes (Li 2025; van Vlerken-Ysla et al. 2023). When oxidized lipids are delivered to tumor cells via liposomes, they can improve the susceptibility of tumor cells to peroxidation induced by hydrogen peroxide (H_2_O_2_) (Tomita 2019), suggesting a potential therapeutic avenue. However, lipid hydroperoxides and reactive aldehydes function as intermediaries within redox signaling pathways that shield cancer cells from oxidative stress-induced cytotoxicity, thereby ultimately conferring resistance to the cells against therapies targeting lipid peroxidation (BorovićŠunjić et al. 2024). This signaling role, integral to understanding the complex interplay in the TME, necessitates further investigation.

## The effect of Lipid peroxidation on immune cells

Given that activated mitochondria cannot metabolize long-chain dicarboxylic acids or very-long-chain fatty acids (VLCFAs) – substrates are exclusively processed by peroxisomal β-oxidation (Tahri-Joutey 2021). It is proposed that these peroxisome-dependent long-to-very-long-chain PUFAs could accumulate in TME and its cells (van Vlerken-Ysla et al. 2023).Immune and stromal cells in the TME also exhibit enhanced lipid synthesis and storage (Jin et al. 2023). Moreover, ferroptosis, which occurs in tumor cells, can spread to neighboring cells via the paracrine release of lipid peroxides (Nishizawa 2021). These dynamics highlight the susceptibility of immune cells to lipid peroxidation. The influence of lipid peroxidation on immune cells within the TME primarily emanates from two sources: first, lipid peroxidation products such as aldehydes and oxidized lipids, predominantly originating from tumor cells, and second, intracellular lipid peroxidation like ferroptosis occurring within the immune cells themselves.

### Neutrophils/PMN-MDSCs

Myeloperoxidase (MPO) is involved in lipid peroxidation within neutrophils. Neutrophils equipped with granules containing MPO can infiltrate the TME, leading to lipid peroxidation and subsequent ferroptosis by increasing intracellular ROS levels (Yee 2020). Polymorphonuclear myeloid-derived suppressor cells (PMN-MDSCs), which are pathologically activated neutrophils, undergo a transition from their normal state under the influence of ferroptosis (Kim 2022). Compared with classical neutrophils, PMN-MDSCs express more S100 A8 and S100 A9, which can activate MPOs in a paracrine and autocrine manner, generating oxidized or hydrolyzed phospholipids that can wake up dormant tumor cells (Perego 2020).

PMN-MDSCs are more susceptible to ferroptosis than neutrophils in the spleen or bone marrow because of the intracellular redox imbalance caused by ROS/reactive nitrogen species (RNS) -generating machinery (Kim 2022). Ferroptosis of PMN-MDSCs leads to intracellular accumulation of oxidized arachidonic acid (AA)-phosphatidylethanolamine (PE) in T cells, limiting their proliferation and function (Kim 2022). Interestingly, lipid peroxidation products generated by PMN-MDSCs through MPO ferroptosis also induced the immunosuppressive function and ferroptosis of macrophages. Compared with Lewis lung cancer (LLC-WT) mice, there was less accumulation of oxidized phospholipids in ferroptotic PMN-MDSCs and a decrease in ferroptosis-associated lipid signals (FALIS) and PGE2 in TAMs of MPO-knockout (KO) mice (Kim 2022). Similarly, PMN-MDSCs can deliver oxidized lipids accumulated by MPO to dendritic cells (DCs), inhibiting their cross-presentation (Ugolini 2020).

Lipid peroxidation products also influence the immunosuppressive N2 phenotype in neutrophils. For example, 4-HNE recruits neutrophils and impairs their metabolic processes by forming intracellular protein adducts (Chacko 2016). This disrupts the glycolytic pathway and inhibits oxidative burst and phagocytosis activities of neutrophils (Chacko 2016). ACR, a lipid peroxidation product generated by hypoxic glioma cells, drives neutrophil reprogramming towards the pro-tumoral N2 phenotype through AKT phosphorylation downregulation, consequently attenuating their anti-tumor functions (Tsai 2023). In the chronic inflammatory environment, ACR enhances the production and activity of enzymes such as matrix metalloproteinase- 9 (MMP- 9) and prolyl endopeptidase, which are pivotal in collagen degradation (Noerager 2015). This enzymatic action contributes to tumor metastasis and extracellular matrix remodeling. Therefore, lipid peroxidation in PMN-MDSCs/neutrophils amplifies their immunosuppressive functions and facilitates tumor progression. However, ACR can upregulate Toll-like receptor 4 (TLR4) expression on the surface of granulocytes, stimulating ROS production via activation of NADPH oxidase (NOX), potentially contributing to tumor regression (Jaganjac et al. 2019).

**Fig. 1:**
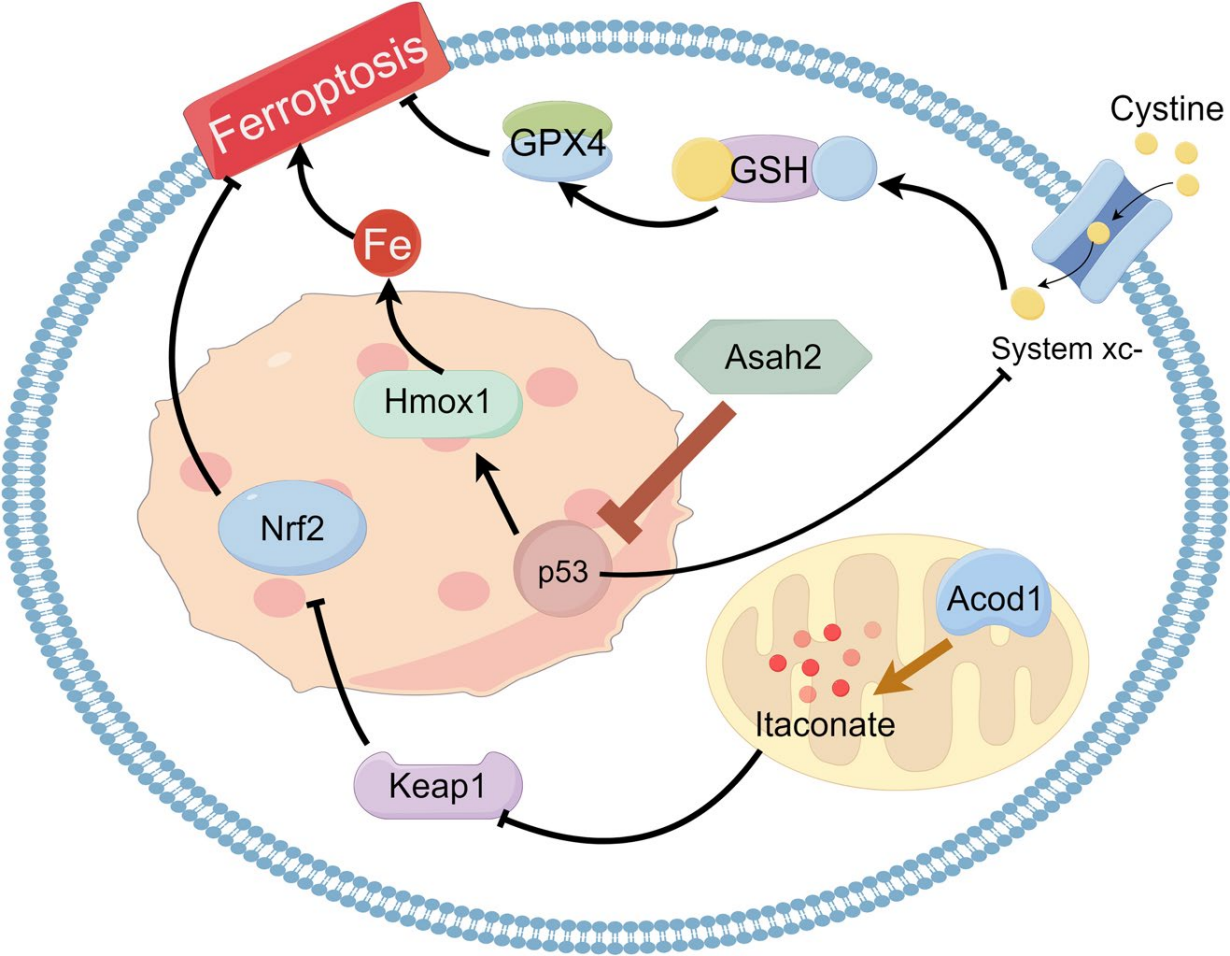
The mechanism of PMN-MDSCs/TINs resistance to ferroptosis. TINs upregulate Acod1 to produce itaconates, which can inhibit Keap1 and activate Nrf2 signaling. Nrf2 is a core transcription factor for antioxidant activity and can prevent ferroptosis through various mechanisms. Similarly, upregulated Asah2 in MDSC suppresses ferroptosis through p53 inhibition. P53 can inhibit SLC7 A11 of Xc.^−^ to suppress the GSH synthesis. P53 can also increase the intracellular iron load through Hmox1. Both pathways p53 activated contribute to the ferroptosis. Acod1: aconitate decarboxylase 1; Asah2: acylsphingosine amidohydrolase; GSH: glutathione; Hmox1: heme-oxygenase 1; Keap1:Kelch-like ECH-associated protein 1;PMN-MDSCs: polymorphonuclear myeloid-derived suppressor cell; SLC7 A11:solute carrier family 7 member 11;TIN: tumor-infiltrating neutrophils;Nrf2: nuclear factor erythroid 2-related factor 2.(By figdraw.com)

Tumor-infiltrating neutrophils (TIN) exhibit resistance to ferroptosis, potentially through the upregulation of aconitate decarboxylase 1 (Acod1), which produces itaconates and activates the Nrf2 pathway (Zhao 2023). Similarly, MDSC resists ferroptosis through the upregulation of N-acylsphingosine amidohydrolase (Asah2). The blockade of Asah2 results in enhanced p53 stability, which mediates ferroptosis of MDSC via excess Fe^2+^ generation and inhibition of glutathione (GSH) synthesis** (**Fig. 1**) **(Zhu et al. 2021). A recent study showed that p53 can induce ferroptosis independent of the GSH/glutathione peroxidase 4 (GPX4) axis by downregulating vitamin K epoxide reductase complex subunit 1 like 1 (VKORC1L1), which prevents the conversion of vitamin K to its antioxidant form, vitamin K hydroquinone (VKH2) (Yang et al. 2023). This mechanism might play a role in the ferroptosis of PMN-MDSCs induced by Asah2 blockade (Zhu 2021).

Neutrophil Extracellular Traps (NETs), mesh-like structures composed of DNA—histone complexes and proteins released by activated neutrophils, play an important role in tumor metastasis (Hu et al. 2023).. In triple-negative breast cancer, NETs contribute to GPX4-mediated resistance to ferroptosis (Yao 2023), possibly acting as a negative feedback mechanism to inhibit ferroptosis of neutrophils surrounding tumor cells through the propagation of ferroptosis. Oxidized phospholipids, major products of lipid peroxidation, have been shown to facilitate NOX phosphorylation and subsequent ROS generation, leading to NETs secretion (Awasthi 2016). Moreover, ACR is known to promote both the chemotaxis of neutrophils and the secretion of NETs (Arumugam et al. 2017). Sulfasalazine (SSZ)-induced phospholipid peroxidation stimulates NET secretion (Yotsumoto 2017)and treatment with the ferroptosis inhibitor Fer- 1 inhibits fluoride-induced NETs release (Wang 2023). In metabolic-associated steatohepatitis (MASH), HMGB1 acts as a damage-associated molecular pattern (DAMP) released by ferroptotic cells, capable of recruiting neutrophils and promoting the formation of NETs (Lv 2023). However, the relationship between HMGB1 and NETs in cancer remains to be further explored. Given the pivotal role of PMN-MDSCs in lipid peroxidation-mediated immunosuppression and the resistance of neutrophils/PMN-MDSCs to ferroptosis, these cells pose a significant challenge in combining ferroptosis induction with immune checkpoint inhibition in cancer therapy. Ferroptosis-induced immunogenic cell death can activate CD8 + T cell-mediated antitumor responses (Lin et al. 2024). However, this beneficial effect is counteracted by the concurrent accumulation of PMN-MDSCs (Conche 2023). Depletion of PMN-MDSCs through CXCR2 inhibition significantly potentiates the therapeutic efficacy of anti-PD- 1/ferroptosis inducer combination therapy in HCC models driven by Nras^G12 V^ and *myr*AKT mutations (Conche et al. 2023).

### Macrophages

Macrophages possess the capability to engulf ferroptotic tumor cells in a TLR2-dependent manner, a process that significantly aids in the therapeutic approach of tumor ferroptosis (Luo 2021). 1-steaoryl- 2–15-HpETE-sn-glycero- 3-phosphatidylethanolamine (SAPE-OOH) on the surface of ferroptotic cells can act as an"eat me"signal (Luo 2021). However, the phospholipid peroxidation within macrophages results in the proteolytic degradation of TLR2, which impairs their ability to engulf ferroptotic tumor cells and contributes to the development of tumor resistance to ferroptosis therapy. The TLR2 agonist, SMU-Z1, has demonstrated the potential to enhance the phagocytic capacity of macrophages by upregulating TLR2 expression. This intervention significantly amplifies the efficacy of ferroptosis-based anti-tumor treatments (Luo 2021; Luo 2024).

Lipid peroxidation significantly influences macrophage polarization, predominantly leading to a proinflammatory phenotype (Xiao et al. 2024). The suppressor of cytokine signaling 1 (SOCS1), a ferroptosis driver, correlates with M1 macrophage infiltration, whereas M2 macrophages are associated with ferroptosis suppressors (Hu 2021). Xc-, a cystine/glutamate transporter comprising the subunits SLC7 A11 and SLC3 A2, plays a pivotal role in delivering cystine into cells for GSH synthesis, thereby conferring resistance to ferroptosis (Chen 2021) (Box 2). The knockdown of XCT which encodes SLC7 A11, can induce M1 polarization by interfering with the SOCS3-STAT6-PPARγ pathway (Tang 2023).

Lipid peroxidation products also contribute to the M1 polarization of macrophages. Oxidized 1-palmitoyl- 2-arachidonyl-phosphatidylcholine (OxPAPC) is a mixture of full-chain and truncated oxidized species, and its pro-inflammatory effects have been extensively demonstrated in atherosclerosis (Di Gioia and Zanoni 2021). OxPAPC reprograms the metabolism of macrophages from glycolysis to mitochondrial respiration, enhancing IL- 1β production and leading to a hyperinflammatory state in macrophages (Di Gioia 2020). OxPAPCs also trigger IL- 1β production through activation of the nod-like receptor protein 3 (NLRP3) inflammasome (Yeon et al. 2017). Epoxyketooctadecenoic acid (EKODE) was markedly increased in the azoxymethane (AOM)/dextran sodium sulfate (DSS)-induced CRC mouse model, activating the NF-κB and JNK pathways in macrophages (Lei et al. 2021). In addition to non-enzyme-dependent lipid peroxidation, macrophages can produce HNE via COX2. HNE activates PKCβ I and II, upregulating monocyte chemoattractant protein 1 (MCP1) to recruit more monocytes (Nitti 2002). These monocytes differentiate into pro-tumor TAMs within the TME (Xu et al. 2021). HNE also impedes macrophage proptosis (Box 1) (Hsu 2022). All of these mechanisms contribute to the accumulation of pro-inflammatory macrophages within the TME.

**Fig. 2:**
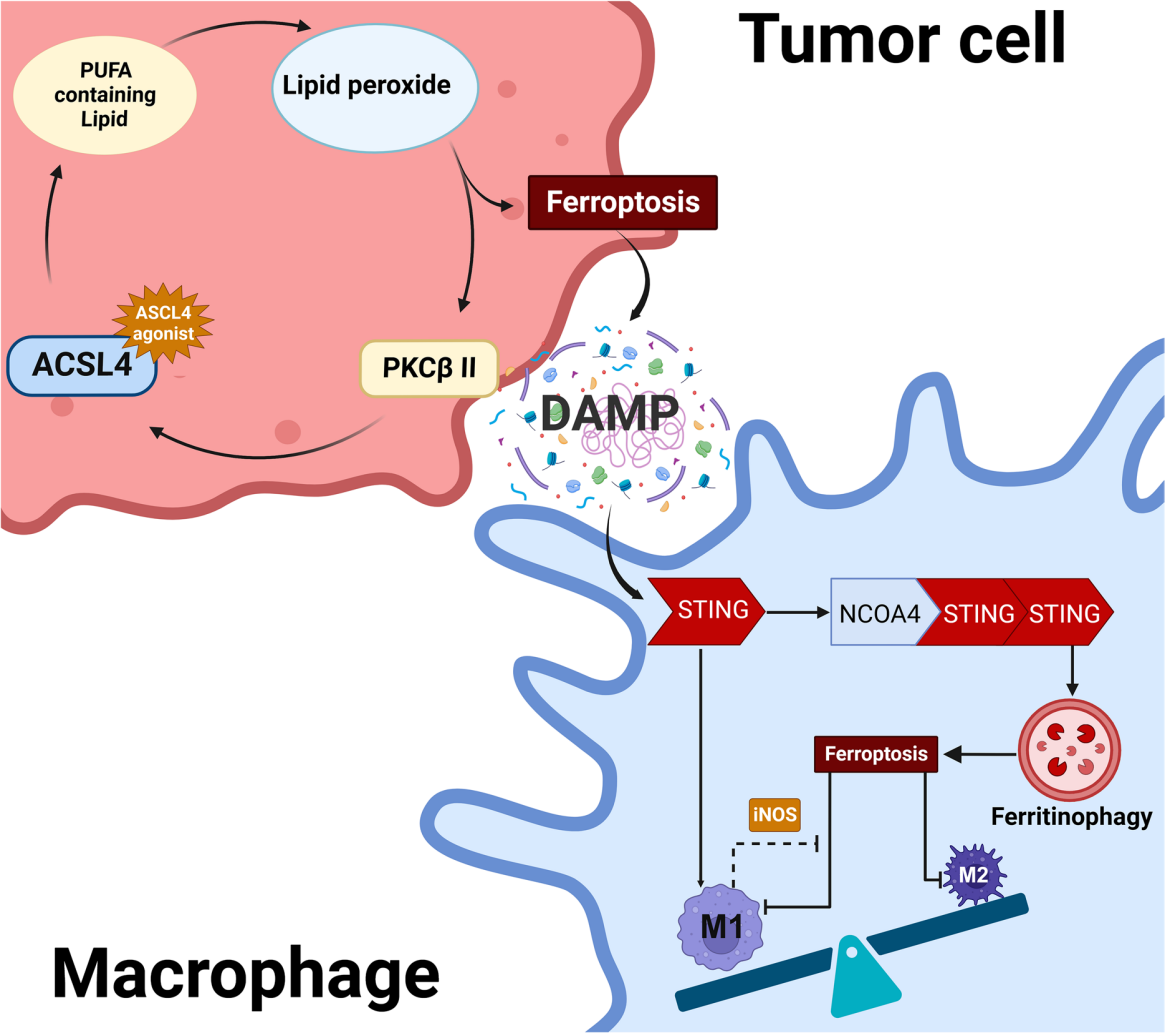
ACSL4 activation induces tumor cell ferroptosis and polarizes macrophages to M1 phenotype. ACSL4 incorporates PUFA into membrane lipids to induce the production of lipid peroxide. Lipid peroxide leads to the ferroptosis of tumor cells and activates PKCβII to promote ACSL4 activation, which is positive feedback. DAMPs from ferroptotic tumor cells can activate STING to skew macrophages to the M1 phenotype. STING can also promote ferritinophagy to induce ferroptosis of macrophages. Due to the high expression of iNOS in M1, M1 exhibits higher resistance to ferroptosis compared with M2. As a result, the ACSL4 agonist promotes the M1 polarization of macrophages. ACSL4: acyl-CoA synthetase long-chain family member 4; iNOS: inducible nitric oxide synthase. (Created with BioRender.com)

The enzyme acyl-CoA synthetase long-chain family member 4 (ASCL4) transforms PUFA, especially AA, into PUFA-CoA, which contributes to the incorporation of PUFA into phospholipids and promotes ferroptosis (Box 2). This process, facilitated by ACSL4, triggers lipid peroxidation and consequent ferroptosis in tumor cells, with a positive feedback loop involving PKCβII-mediated phosphorylation of ACSL4 (Zhang 2022). DAMPs in ferroptotic tumor cells bind to advanced glycosylation end-product specific receptors (AGER), stimulating the production of tumor necrosis factor (TNF) (Wen et al. 2019). These DAMPs can also activate cGAS-STING signaling (Dai 2020), and STING further triggers ferroptosis in macrophages through enhanced ferritinophagy (Box 1) mediated by nuclear receptor coactivator 4 (NCOA4) (Wu 2022). Macrophages can undergo polarization from the M2 to M1 phenotype through ACSL4-induced ferroptosis and STING activation, suggesting that ACSL4 activation is a promising strategy for macrophage reprogramming in cancer therapy**(**Fig. 2**)**(Cao 2024;, Chen 2023).

Although numerous studies have shown that lipid peroxidation contributes to the pro-inflammatory phenotype of macrophages, it occasionally induces an M2 phenotype. The concentration of lipid peroxidation-derived aldehydes is pivotal to this dichotomy. A high concentration of EKODE typically activates the Nrf2 signaling pathway and inhibits NF-κB, which may contribute to M2 macrophages (Lei et al. 2021). This dual activity highlights the complexity of lipid peroxidation products in modulating immune responses (Martín-Sierra et al. 2019). Macrophages can internalize KRAS^G12D^ exosome released from ferroptotic cancer cells via AGER. This uptake increases fatty acid oxidation (FAO) and M2 polarization of macrophages (Dai et al. 2020), which is typically associated with anti-inflammatory and tissue-repair functions. Notably, M1 macrophages are more resistant to ferroptosis than M2 macrophages, largely because of elevated levels of inducible nitric oxide synthase (iNOS) (Kapralov 2020). However, this resistance can be mitigated by oxidized phosphatidylcholine (OxPC) produced during ferroptosis, which inhibits iNOS production by preventing NF-κB binding to the iNOS promoter (Friedl et al. 2006).While M1 macrophages are generally considered to exert anti-tumor effects in the TME, they can paradoxically facilitate tumorigenesis by releasing inflammatory mediators (Dai 2020; Su 2023; Taniguchi and Karin 2018). Consequently, the dynamic balance between M1 and M2 macrophages is a critical determinant of tumor progression. Understanding and modulating this balance could provide insights into the potential therapeutic strategies for cancer treatment.

### Dendritic cells

Large lipid bodies (LBs) within tumor-associated DCs contain electrophilic oxidatively truncated lipids that have been shown to interact with heat shock protein 70 (Hsp70). This interaction disrupts the transport of peptide–MHC class I (pMHC I) complexes to the cell surface, consequently impeding the antigen-presenting capabilities of DCs. Considering the limited capacity of DCs to oxidize intracellular lipids, these truncated lipids are likely derived from the TME (Ramakrishnan 2014;, Veglia 2017), and lipid peroxidation in tumor cells is a significant source. DCs exhibit stage-dependent functional responses to engulfed ferroptotic tumor cells, shaped by lipid peroxidation dynamics. While early ferroptotic cells (rich in highly reactive electrophilic lipids) suppress DC maturation, later-stage ferroptotic cells enhance surface costimulatory markers (CD86, CD40, MHC II). Crucially, all engulfed ferroptotic cells impair DC antigen presentation—a defect driven by intracellular lipid droplet accumulation (Wiernicki 2022). Consequently, loading tumor vaccines with ferroptotic cells fails to elicit effective immune protection, suggesting that the hypothesized increased immunogenicity from ferroptosis may not stem from DCs, but rather from other antigen-presenting cells like macrophages (Efimova 2020; Wiernicki 2022). 15-LOX induces ferroptosis by converting AA-PE or adrenic acid-containing phosphatidylethanolamine (AdA-PE) into ferroptotic signals (D'Herde and Krysko 2017). 15-LOX can also produce OxPC, which inhibits DC maturation (Rothe 2015). OxPE, a primary oxidized phospholipid in ferroptosis execution (Demuynck et al. 2021), binds to milk fat globule-EGF factor 8 (MFG-E8), which is essential for phagocytosis of apoptotic cells by inflammatory monocytes (Uderhardt 2012). Since DCs also express MFG-E8, OxPE may interfere with their ability to engulf apoptotic tumor cells. OxPAPC generally promotes inflammation in myeloid cells. However, it can prevent DCs from secreting IL- 12 depending on the cpoxycyclopentenone structure of OxPAPC, which is essential for skewing CD4 + T cells towards a Th1 phenotype and facilitating an anti-tumor response (Bretscher 2015). Furthermore, 4-HNE, a product of lipid peroxidation, can induce endoplasmic reticulum (ER) stress in DCs. Stress sensors like inositol-requiring protein 1a (IRE1α), detect this stress and facilitate the generation of functional X-box binding protein 1 (XBP1), which in turn promotes triglyceride biosynthesis, leads to lipid accumulation, and dampens the antigen-presenting function of DCs (Cubillos-Ruiz 2015). DC ferroptosis also appears to limit the secretion of pro-inflammatory factors and expression of MHC I (Hu 2021). However, lipid peroxidation within the endosomal membranes can promote the release of antigens from the endosomal lumen, thereby improving the cross-presentation capabilities of DCs (Calzada-Fraile 2023; Dingjan 2016). Therefore, the spatial heterogeneity of lipid peroxidation within DCs intricately dictates their function and influences their role in the immune response to tumors.

### T lymphocytes

Recent evidence has underscored the significance of ferroptosis in bolstering the anti-tumor roles of CD8 + T cells and enhancing the efficacy of anti-PD- 1 therapy (Wang 2019). Interferon-γ (IFN-γ) released from T cells plays a pivotal role in this process by downregulating the expression of SLC3 A2 and SLC7 A11 in tumor cells (Wang 2019), which are crucial for GSH synthesis and ferroptosis resistance. IFN-γ also promotes expression of interferon regulatory factor1 (IRF1). IRF1, in turn, binds to the promoter region of ACSL4 (Liao 2022), upregulating its expression, thereby facilitating lipid peroxidation and ferroptosis in tumor cells.

Lipid peroxidation products, HNE enhances the expression of Fas ligand (FasL) on T cells, although this effect can be blocked by GSH overexpression. MDA reduces the viability of T cells, particularly under conditions of GSH depletion (Chang et al. 2008). Lipid peroxidation products also affect CD4 + T cell differentiation (Martín-Sierra et al. 2019). MDA-adducted mouse serum albumin (MSA) and HNE–MSA can promote CD4 + T cell proliferation and bias them towards a Th1 phenotype (Wang et al. 2008). Conversely, MDA-laminin adducts have been shown to suppress the generation of T regulatory cells (Tregs) (Duner 2010). OxPAPCs can further skew the differentiation of Tregs towards Th1 cells via IFN-γ signaling (Appleton et al. 2023). Within the TME, Tregs typically express high levels of GPX4 to counteract intracellular lipid peroxidation. GPX4-deficient Tregs accumulate lipid peroxides and produce IL- 1β, which activates DCs and CD8 + T cells, thereby fostering anti-tumor immunity. IL- 1β also amplifies the Th17 response (Xu 2021). In summary, lipid peroxidation modulates the T cell landscape by enhancing Th1 and Th17 differentiation while diminishing Treg populations. Th1 cells exert anti-tumor effects and Th17 cells have been implicated in promoting angiogenesis and tumor progression (Lança and Silva-Santos 2014). Th17 cells are also associated with improved survival outcomes in patients with ovarian cancer and esophageal squamous cell carcinoma (Lança and Silva-Santos 2014). This dichotomy underscores the complexity of T cell differentiation influenced by lipid peroxidation products and highlights the variable impact of such processes on patient prognosis, reflecting the diversity of the TME.

The aforementioned findings reveal that T cell-mediated lipid peroxidation and its metabolic products generally bolster anti-tumor immune responses. However, intracellular lipid peroxidation within T cells themselves may exert detrimental effects, thereby counteracting the beneficial outcomes of T cell-mediated anti-tumor immunity (Lin et al. 2024).CD36, a scavenger receptor integral to lipid metabolism, allows CD8 + T cells to internalize oxidized low-density lipoprotein (Ox-LDL). This uptake triggers intracellular lipid peroxidation, leading to CD8 + T-cell dysfunction (Xu 2021). Additionally, CD36 facilitates the uptake of fatty acids, exacerbates intracellular lipid peroxidation, and pushes T cells toward ferroptosis, which is marked by reduced production of essential cytotoxic cytokines. Inhibition of ferroptosis can bolster the anti-tumor activities of CD8 + T cells, and the expression of CD36 correlates with poorer patient prognoses (Ma 2021). CD36 can also prevent apoptosis of Tregs by assisting their metabolic adaptation to the TME. Intriguingly, Treg depletion caused by CD36 deficiency does not affect maintaining peripheral immune homeostasis. Furthermore, anti-CD36 mAbs can amplify the efficacy of anti-PD- 1 therapy (Wang 2020). This suggests a potential strategy for the blockade of CD36 in combination with immunotherapies such as chimeric antigen receptor T-cell (CAR-T) therapy and immune checkpoint inhibitors (ICI), warranting further investigation.

IFN-I is typically regarded as an anti-tumor component of the TME. However, chronic IFN-I exposure can disrupt the redox balance in T cells by downregulating glutathione and facilitating T cells to take up long-chain fatty acids but inhibiting FAO, leading to lipid accumulation and peroxidation, which ultimately results in CD8 + T cell exhaustion (Chen 2022). Chronic exposure to IFN-I can also upregulate PD-L1 expression on DCs and mediate the resistance of tumor cells to radiotherapy and chemotherapy (Bazhin et al. 2018; Weichselbaum 2008). This phenomenon suggests that prolonged IFN-I exposure may inadvertently dampen CD8 + T cell responsiveness and hinder their anti-tumor capacity. Additionally, T-IFNAR1^−/−^tumor-bearing mice exhibited a better response to anti-PD- 1 than WT mice (Chen 2022), which also indicates that IFN-I impedes the efficacy of Immune checkpoint inhibition. Interestingly, CD8 + T cells are more prone to ferroptosis induced by GPX4 inhibition than tumor cells (Drijvers 2021), suggesting that direct induction of ferroptosis within the TME may be less than ideal. Recently, Yang et al. identified that low expression of apolipoprotein L3 (APOL3) is associated with poor patient outcomes and enhanced metastasis in CRC. APOL3, by targeting L-lactate dehydrogenase A (LDHA) for degradation through ubiquitination, can potentiate tumor ferroptosis and the cytotoxic potential of CD8 + T cells. Hence, overexpression of APOL3 may serve as an effective strategy to selectively induce ferroptosis in tumor cells, sparing immune cells within the TME (Lv 2023).

### Nature killer cells

The majority of natural killer (NK) cells in gastric cancer retain their intrinsic anti-tumor activities and play an essential role in immunotherapy (Li 2015). Therefore, it is crucial to prevent ferroptosis in NK cells to maintain their efficacy in cancer therapies. L-kynurenine (L-KYN) exerts immunosuppressive effects via the aryl hydrocarbon receptor (AHR). Notably, gastric cancer cells generate L-KYN to trigger ferroptosis in NK cells via an AHR-independent pathway (Cui 2023). Cancer-associated fibroblasts (CAFs), which protect tumor cells from ferroptosis (Qi 2023; Yao 2023), paradoxically promote ferroptosis in NK cells. CAFs can enhance iron content in the TME by upregulating iron export proteins ferroportin1 (FPN1) and hephaestin (HEPH). Additionally, CAFs secrete a follistatin-like protein (FSTL) that interacts with disco interacting protein 2 homolog A (DIP2 A) to promote ferritinophagy (Yao 2023). Both pathways can increase the labile iron pool within NK cells to induce ferroptosis. Lipid peroxidation also has detrimental effects on NK cells, impairing their glucose metabolism and production of IFN-γ, especially under oxidative stress conditions prevalent in the TME (Poznanski 2021). Collectively, these findings indicate that lipid peroxidation within the TME can significantly reduce the anti-tumor functions of NK cells, highlighting the need for strategies to mitigate this effect and enhance the efficacy of NK cell-based cancer therapies.

### Stroma cells

Stromal cells, comprising CAFs, mesenchymal stem cells, adipocytes, endothelial cells, and pericytes, dynamically interact with tumor and immune cells to modulate tumor initiation, progression, and metastasis (Zhao 2023). Despite their critical involvement, research on lipid peroxidation in stromal cells remains limited. A recent study reveals that CD36 + CAFs internalize Ox-LDL to trigger lipid peroxidation, subsequently secreting macrophage migration inhibitory factor (MIF) to recruit MDSCs, thereby establishing an immunosuppressive TME (Zhu 2023).In recurrent glioblastoma, NOX4-mediated endothelial ferroptosis is upregulated, yet its underlying mechanisms require further investigation (Liang et al. 2024). Intriguingly, a recent study demonstrated that sublethal ferroptosis promotes endothelial cell proliferation, migration, and vessel-like structure formation, while concurrently enhancing endothelial adhesion and trans-endothelial migration of tumor cells (Lopes-Coelho 2021). However, endothelial cell ferroptosis is one of the mechanisms by which the anti-angiogenic drug apatinib exerts its anti-tumor effects (Wang 2024). These findings underscore the necessity for cautious evaluation of endothelial cell responses when developing ferroptosis-targeted anticancer therapies.

Stromal cells further regulate tumor ferroptosis to mediate chemotherapeutic resistance (Cui et al. 2024). Among stromal components, CAFs have garnered significant attention. CAFs promote ferroptosis resistance in pancreatic ductal adenocarcinoma by supplying cysteine to fuel GSH biosynthesis in tumor cells (Zhu 2024). CAF-derived exosomal miR- 432 - 5p targets cation transport regulator homolog 1 (CHAC1), reducing GSH consumption and ferroptosis, thereby conferring docetaxel resistance on prostate cancer cells (Zhao 2024). Conversely, CAF-derived exosomal DACT3-AS1 enhances oxaliplatin sensitivity in cancer cells through sirtuins 1 (SIRT1)-mediated ferroptosis (Qu 2023). Adipocytes demonstrate context-dependent regulatory duality. Their exosomal microsomal triglyceride transfer protein (MTTP) upregulates GPX4 and XCT to inhibit ferroptosis in colorectal cancer (Zhang 2022), while adipocyte-secreted oleic acid attenuates lipid peroxidation and ferroptosis in triple-negative breast cancer cells in an ACSL3-dependent manner (Xie 2022). However, high concentrations of free fatty acids released by adipocytes paradoxically induce ferroptosis in myeloma cells (Panaroni 2022). This functional plasticity highlights the multifaceted role of stromal cells in shaping ferroptotic responses and further research is required to clarify their therapeutic implications in ferroptosis-based cancer treatment.

## Conclusion and Perspective

**Fig. 3:**
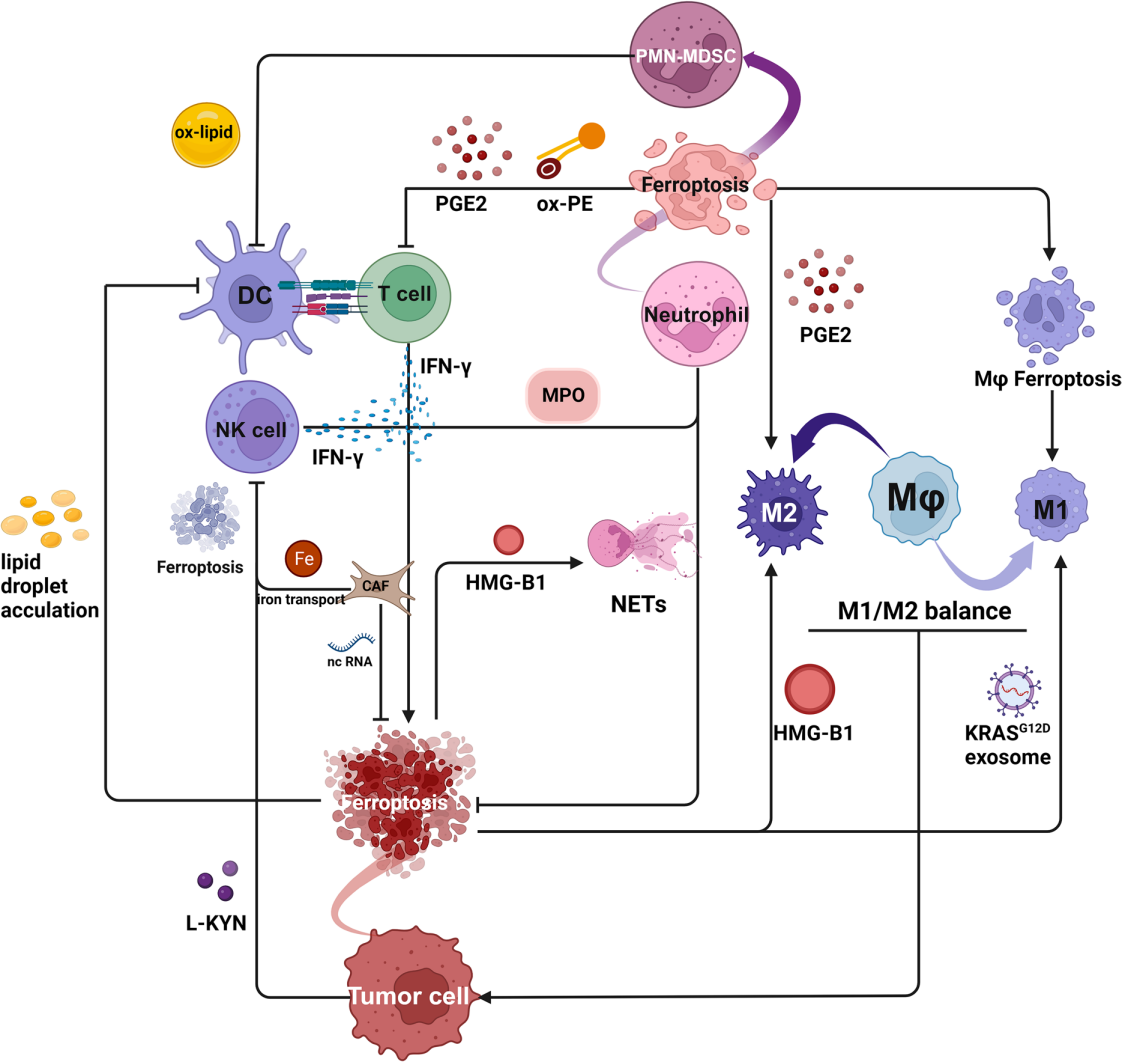
Ferroptosis mediated cell crosstalk between immune cells and tumor cells. The susceptibility of tumor cells to lipid peroxidation makes ferroptosis an anti-tumor method. IFN-γ from T cells and NK cells as well as MPO-containing granules from neutrophils can induce tumor cells ferroptosis. However, ferroptotic tumor cells can release HMG-B1 to promote the NETs secretion to inhibit ferroptosis. CAFs can also prevent tumor cell ferroptosis via non-coding RNA (ncRNA) including miR- 3173 - 5p, miR- 522 and DLEU1. Besides, CAFs can increase the iron labile pool of NK cells to induce ferroptosis. Ferroptosis can also foster a tumor-promoting milieu. Ferroptosis in neutrophils can induce their pathological phenotype PMN-MDSC, and subsequent PMN-MDSCs ferroptosis inhibits the function of macrophages, DCs and T cells, which indicates PMN-MDSCs, play a pivotal role in lipid peroxidation-mediated immunosuppressive environment. Ferroptotic products from PMN-MDSCs and tumor cells promote macrophage polarization to both M1 and M2, which might lead the balance of inflammation and immunosuppression to facilitate tumor progression. CAF: cancer-associated fibroblasts; DC: dendritic cell; HMG-B1: high-mobility-group B1; L-KYN: L-kynurenine; MPO: myeloperoxidase; NET: neutrophil extracellular trap; OxPE: oxidized phosphatidylethanolamine; PGE2: prostaglandin E2; PMN-MDSC: polymorphonuclear myeloid-derived suppressor cell. (Created with BioRender.com)

The susceptibility of tumor cells to lipid peroxidation due to oxidative stress and lipid accumulation in the TME has rendered ferroptosis induction a focal point in anti-tumor strategies. Nonetheless, lipid peroxidation also affects immune cell function, notably causing neutrophils to adopt an immunosuppressive PMN-MDSC phenotype that dampens the responsiveness of macrophages, DCs, and T cells. This transformation underscores the central role of PMN-MDSCs in immunosuppression mediated by lipid peroxidation** (**Fig. 3**)**.

Lipid peroxidation is implicated in promoting NETs secretion and inhibiting the neutrophil oxidative burst, potentially as a defensive feedback mechanism that allows tumor cells toSREBP to excess oxidative stress. Paradoxically, ferroptotic cells generate inflammatory lipid mediators, such as hydroxyeicosatetraenoic acid (HETE), LTB4, and PGE2 (FriedmannAngeli et al. 2019; Johnson et al. 2020), contributing to TME inflammation. However, high concentrations of lipid peroxidation products may drive M2 polarization, tempering macrophage-mediated inflammation and oxidative stress, while the predominance of either outcome depends on the level of ROS, lipid type, and histological context of the tumor.

Moreover, lipid peroxidation can impair the antigen-presenting function of DCs and the cytotoxic actions of CD8 + T cells, thereby eroding adaptive immunity. However, IFN-γ-induced ferroptosis supports the anti-tumor activity of CD8 + T cells. Although PMN-MDSCs have not been directly implicated in the lipid peroxidation-mediated inhibition of NK cells, both CAFs and tumor cells within the TME can trigger ferroptosis in NK cells. In contrast, CAFs may protect tumor cells from ferroptosis, underscoring their critical role in impeding NK cell-mediated tumor suppression.

Recent study by Kim et al. have delineated the temporal separation of immunosuppression from actual cell death in neutrophil ferroptosis (Kim 2022), which is attributable to lipid peroxidation and its antecedent products. Extending this theory to all immune cells within the TME, it is apparent that lipid peroxidation and its products foster an inflammatory environment via M1 polarization and release of lipid mediators and DAMPs. Conversely, they also thwart anti-tumor responses by interfering with the functions of DCs, NK cells, and CD8 + T cells, thereby facilitating tumor progression. Lipid peroxidation-mediated cell crosstalk**(**Fig. 3**),** primarily involving PMN-MDSCs, poses a significant challenge to effectively harness ferroptosis in cancer therapy.

**Fig. 4:**
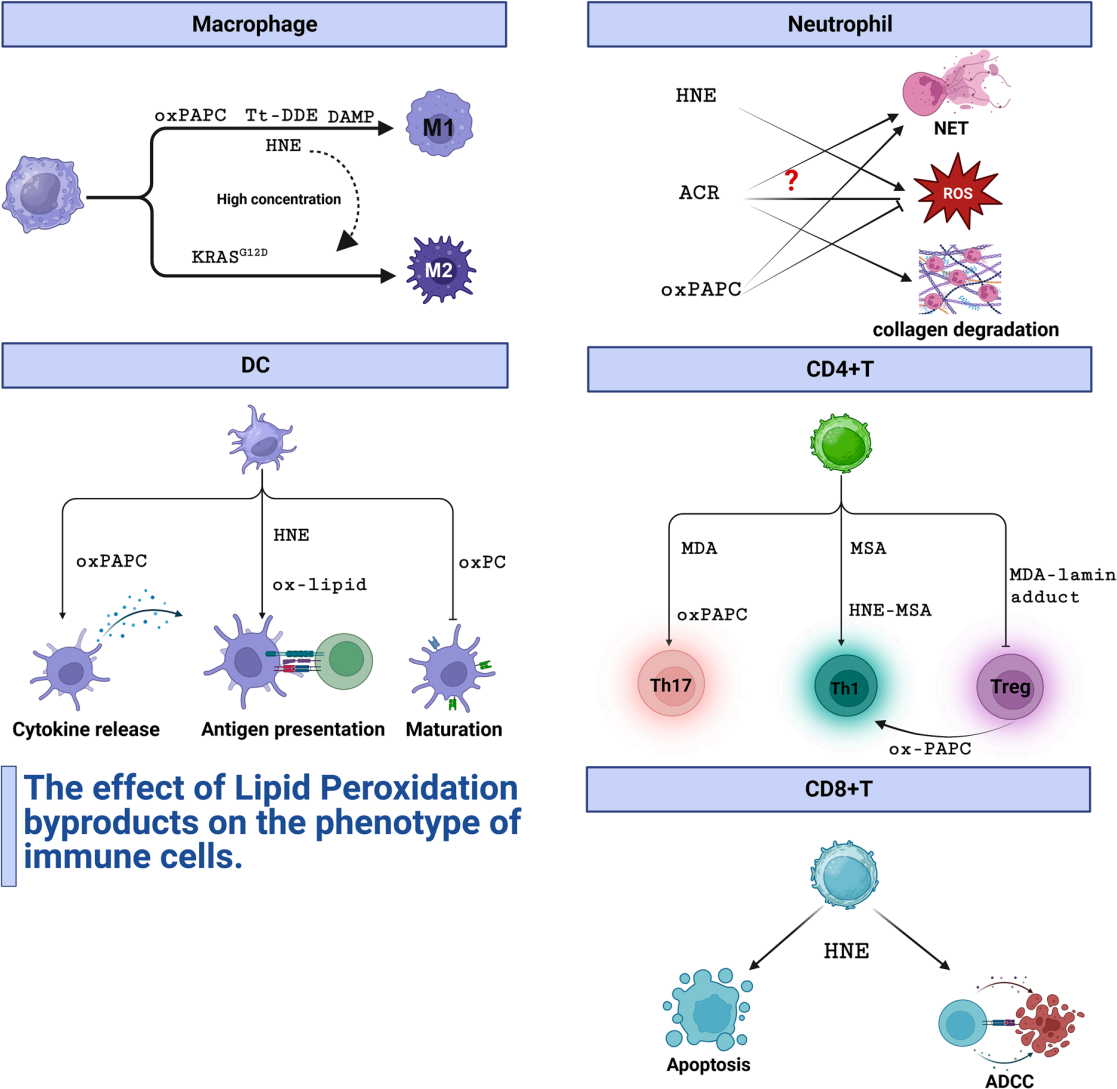
The effect of lipid peroxidation products on the phenotype of immune cells OxPAPC,Tt-DDE,DAMPs and low concentration of HNE polarize macrophages to M1 phenotype, while high concentration of HNE and KRAS.^G12D^ exosomes skew macrophages to M2 phenotype. ACR and OxPAPC stimulate the secretion of NETs in neutrophils; ACR can also promote the enzyme secretion of neutrophils for collagen degradation. The role of lipid peroxidation products in ROS release is complicated. HNE can promote ROS release of neutrophils while OxPAPC inhibits ROS generation. ACR can suppress ROS secretion directly but can also increase ROS production through upregulating TLR4 on cell surface. Lipid peroxidation products can also limit DCs’ cytokine secretion, antigen presentation and maturation and promote CD4 + T cells differentiation to Th17 and Th1. MDA-lamin adduct inhibit T cell to differentiate into Tregs and OxPAPC can transform Treg to Th1. HNE induce CD8 + T cell apoptosis. However, it can also enhance their ability of ADCC. ACR: acrolein; ADCC: antibody dependent cell mediated cytotoxicity; DAMP: damage-associated molecular pattern; HNE: hydroxynonenal; OxPAPC: oxidized 1-palmitoyl- 2-arachidonyl-phosphatidylcholine; Tt-DDE: 14–3 - 3ζ, trans- 2,4-decadienal;ROS: reactive oxygen species; Treg: T regulatory cell. (Created with BioRender.com)

Numerous studies suggest that ferroptosis can contribute to the therapeutic efficacy of chemotherapy, radiotherapy, and immunotherapy (Zhou 2024). However, the inherent heterogeneity of tumors and their immune microenvironment complicates its role in tumor suppression. The immunosuppressive properties of ferroptosis coupled with inflammatory responses triggered by therapy-induced tissue damage potentially drive tumor progression (Dang 2022; He 2024). Several ongoing clinical trials are currently evaluating the efficacy of ferroptosis inhibitors in cancer treatment**(**Table 1**)**. However, further research is imperative to determine whether their antitumor potential stems specifically from ferroptosis inhibition or involves alternative molecular mechanisms. One of the approaches to resolve the dilemma of applying ferroptosis in cancer treatment is to achieve cell-specific delivery. Nanotherapies targeting tumor cells with precision while sparing the surrounding immune milieu have shown promise in preclinical trials, especially when combined with immunotherapy to induce ferroptosis (Hsieh 2021;Song 2021; Zheng and Guan 2023). (Liu 2022; Jiang 2021) This synergy between immunotherapy and ferroptosis induction can be groundbreaking. However, research often overlooks the role of low-concentration lipid peroxidation products like HNE in vitro **(**Fig. 4**)**, because its consistency with ferroptosis might mask their potential adverse effects on immune cells. Given the ability of products especially aldehydes to diffuse readily and directly impair DNA, leading to tumorigenesis and a tumor-promoting environment, there is a critical need for further preclinical and clinical scrutiny to ascertain whether long-term cancer therapy promotes the formation of new lesions or pre-metastatic niches.

**Table 1:** Clinical trials of lipid peroxidation and ferroptosis antagonist therapies

Drug	Target	Mechanism	Indication	Trail ID	Phase
Vitamin E	RTA/ALOXs	Lipid peroxidation inhibitor	PDAC	NCT01446952	I
			Prostate Cancer	NCT00809458	II
			CRC	NCT00905918	I
			Breast Cancer	NCT01571921	Ia
				NCT04496492	II
Deferoxamine	Iron	Iron chelator	TNBC	NCT05300958	II
			Solid tumors	NCT05184816	I
Deferasirox	Iron	Iron chelator	MDS	NCT00940602	II
Ciclopirox	Iron	Iron chelator	Relapsed or Refractory Hematologic Malignancy	NCT00990587	I
Troglitazone	ACSL4	ACSL4 inhibitor	Liposarcoma	NCT00003058	II
Rosiglitazone	ACSL4	ACSL4 inhibitor	Solid tumor	NCT04114136	II
			Prostate cancer	NCT00182052	III
			Liposarcoma	NCT00004180	II
Pioglitazone	ACSL4	ACSL4 inhibitor	Thyroid Cancer	NCT01655719	II
			NSCLC	NCT01342770	II
NAC	GSH	GSH synthesis regulator	Lymphoma	NCT05081479	I

We retrieved clinical studies where ferroptosis or lipid peroxidation antagonists were used alone to suppress tumors. All the information in this table comes from clinicaltrials.gov and can be retrieved through the Trail ID (Completed on March 15 th,2025). ACSL4: acyl-CoA synthetase long-chain family member 4; CRC: Colorectal cancer; GSH: glutathione; MDS: myelodysplastic neoplasms; NAC: N-acetylcysteine; NSCLC: non-small cell lung cancer; PDAC: pancreatic ductal adenocarcinoma; TNBC: triple-negative breast cancer

Importantly, the impact of these products is contingent on their concentration and the specific macromolecules they target. Therefore, it is imperative to investigate the effects of these products in a specific TME context. Although such studies are scarce **(**Table 2**)**, our review aimed to increase the awareness of the potential side effects associated with prolonged ferroptosis-inducing therapies.

**Table 2:** The mechanism of lipid peroxidation affecting immune cells

Lipid peroxidation products	Immune cell	Mechanism	Function	Ref
4-HNE	Neutrophil	Pleiotropic mechanism	Inhibition of oxidative burst and phagocytosis	(Chacko et al. 2016)
ACR		Inhibiting AKT phosphorylation	Induction of anti-inflammatory phenotype	(Tsai et al. 2023)
		Induce the production and functionality of collagen-degrading enzymes	Collagen degradation	(Noerager et al. 2015)
		Upregulation of TLR4	Stimulating ROS production	(Jaganjac et al. 2019)
		NOX2 and p38MAPK	Stimulating NETs secretion	(Arumugam et al. 2017)
Oxidized phospholipid		TLR2/6-PKC-IRAK-MAPK-NOX	Stimulating ROS production and NETs secretion	(Awasthi et al. 2016)
OxPS	Macrophages	“Eat me” signal	Promoting phagocytosis	(Greenberg et al. 2006; Tyurin et al. 2014)
SAPE-OOH				(Luo et al. 2021)
EKODE (Low concentration)		NF-κB/JNK pathway	Pro-inflammatory phenotype	(Lei et al. 2021)
Tt-DDE				(Wang et al. 2020b)
HNE		Impede the interaction between NLRP3 and NEK7	Inhibition of pyroptosis	(Hsu et al. 2022)
OxPC	DC	Nrf2 signaling	Inhibiting the maturation and activation of DCs	(Rothe et al. 2015)
OxPAPC			Inhibiting the secretion of IL- 12	(Bretscher et al. 2015)
4-HNE		ER stress-triglyceride biosynthesis	Dampen the antigen-presenting function	(Cubillos-Ruiz et al. 2015)
HNE	T cell	Deactivate AKT	Enhanced FasL expression	(Chang et al. 2008)
MDA			Reduce T cell viability	
OxPAPC		IFN signaling	Induce Tregs to differentiate into Th1 phenotype	(Appleton et al. 2023)

*ACR* acrolein, *DC* dendritic cell, *EKODE* epoxyketooctadecenoic acid, *ER* endoplasmic reticulum, *FasL* Fas ligand, *HNE* hydroxynonenal, *IFN* interferon, *TLR* Toll-like receptor, *MDA* malondialdehyde, *NEK7* NIMA-related kinase 7, *NLRP3* nod-like receptor protein 3, *NOX* NADPH oxidase, *Nrf2* nuclear factor erythroid 2-related factor 2, *OxPAPC* oxidized 1-palmitoyl- 2-arachidonyl-phosphatidylcholine, *OxPC* oxidized phosphatidylcholine, *OxPS* oxidized phosphatidylserine, *ROS* reactive oxygen species, *SAPE-OOH* 1-steaoryl- 2–15-HpETE-sn-glycero- 3-phosphatidylethanolamine, *Treg* T regulatory cell, *Tt-DDE* 14–3 - 3ζ, trans- 2,4-decadienal

## Box 1. Terminology and definitions

Lipid Peroxidation: A specific form of radical attack, usually at bis-allylic sites of an unsaturated hydrocarbon chain, which first leads to a carbon-centered radical and then the addition of molecular oxygen to form a peroxyl radical on that carbon. The peroxyl radical can take hydrogen atoms from nearby molecules, initiating a chain reaction that spreads the damage.

Lipoxidation: Covalent reaction between electrophilic lipid products and large molecules, including proteins, DNA, or head groups of phospholipids.

Ferroptosis: a form of iron-dependent cell death driven by unrestricted lipid peroxidation and subsequent plasma membrane rupture.

Pyroptosis: a type of gasdermin-dependent programmed cell death that induces cytoplasmic holes and stimulates the release of various pro-inflammatory and immunogenic mediators upon cell rupture, thereby promoting immune cell activation and accumulation.

Ferritinophagy: The process by which ferritin is recognized by NCOA4 and delivered to the autophagosome.

## Box 2. Lipid peroxidation and ferroptosis

Ferroptosis is a kind of iron-dependent regulated cell death. Excess intracellular iron can induce lipid peroxidation through Fenton reaction and iron-containing enzymes, which destroy the cell membrane and induce cell death (Stockwell 2017). The core mechanism regulating ferroptosis involves the balance between oxidative damage and antioxidant defense (Kuang et al. 2020). The antioxidant enzyme GPX4 plays an important role in repressing ferroptosis relying on GSH and selenium. GSH consists of three amino acids: cysteine, glycine, and glutamic acid, and cysteine availability is the main limiting factor in the synthesis of GSH. System xc^−^can import cystine into the cells for subsequent GSH production. Several non-GPX4 pathways, including the AIFM2–CoQ10 (Bersuker 2019;, Doll 2019), GCH1–BH4 (Kraft 2020)and ESCRT-III (Dai et al. 2020) pathways, can also protect cells from ferroptosis.

PUFAs are the most susceptible to lipid peroxidation. The incorporation of PUFA into the cell membrane can induce ferroptosis, which requires the enzymes ACSL4 and LPCAT3. ACSL4 catalyzes the production of AA-CoA or AdA-CoA, and LPCAT3 promotes their esterification to membrane phospholipids, facilitating lipid peroxidation of lipid bilayers and subsequent cell death (Dixon 2015;, Doll 2017).

## Data Availability

No datasets were generated or analysed during the current study.

## Abbreviations

*AA*: Arachidonic acid
*AADAC*: Arylacetamide deacetylase
*ACADM*: Acyl-coenzyme A dehydrogenase
*Acod1*: Aconitate decarboxylase 1
*ACR*: Acrolein
*ACSL4*: Acyl-CoA synthetase long-chain family member 4
*AdA*: Adrenic acid
*AGER*: Advanced glycosylation end-product specific receptor
*AHR*: Aryl hydrocarbon receptor
*AOM*: Azoxymethane
*APC*: Adenomatous polyposis coli
*APOL3*: Apolipoprotein L3
*ASC*: Apoptosis-associated speck-like protein
*ASCL4*: Acyl-CoA synthetase long-chain family member 4
*Asah2*: Acylsphingosine amidohydrolase
*CAF*: Cancer-associated fibroblast
*CAR-T*: Chimeric antigen receptor T-cell
*CHAC1*: Cation transport regulator homolog 1
*COX*: Cyclooxygenase
*CPT1 A*: Carnitine palmitoyltransferase 1 A
*CRC*: Colorectal cancer
*CXCR2*: C-X-C chemokine receptor
*DAMP*: Damage-associated molecular pattern
*DC*: Dendritic cell
*DHCR7*: 7-Dehydrocholesterol reductase
*DIP2 A*: Disco interacting protein 2 homolog A
*DSS*: Dextran sodium sulfate
*EKODE*: Epoxyketooctadecenoic acid
*EMT*: Epithelial-to-mesenchymal transition
*ER*: Endoplasmic reticulum
*FALIS*: Ferroptosis-associated lipid signals
*FAO*: Fatty acid oxidation
*FasL*: Fas ligand
*FSTL*: Follistatin-like protein
*FPN1*: Ferroportin1
*GPX4*: Glutathione peroxidase 4
*GSDMD*: Gasdermin D
*GSH*: Glutathione
*HCC*: Hepatocellular carcinoma
*HEPH*: Hephaestin
*HHE*: Hydroxy-hexenal
*HIF*: Hypoxia inducible factor
*HMGB1*: High-mobility group protein- 1
*HNE*: Hydroxynonenal
*Hsp*: Heat shock protein
*ICI*: Immune checkpoint inhibitors
*IDO*: Indoleamine 2, 3-dioxygenase
*IFN*: Interferon
*Inos*: Inducible nitric oxide synthase
*IRAK*: Interleukin- 1 receptor-associated kinase
*IRE1α*: Stress sensors likeinositol-requiring protein 1a
*IRF1*: Interferon regulatory factor1
*JAK*: Janus kinase
*Keap1*: Kelch-like ECH-associated protein 1
*KO*: Knockout
*LB*: Large lipid bodies
*LDHA*: L-lactate dehydrogenase A
*LLC*: Lewis lung cancer
*LKB1*: Live kinase B1
*L-KYN*: L-kynurenine
*LOX*: Lipoxygenase
*LPCAT1*: Lysophosphatidylcholine acyltransferase 1
*LTB4*: Leukotriene B4
*MASH*: Metabolic-associated steatohepatitis
*MAPK*: Mitogen-activated protein kinase
*MCP1*: Monocyte chemoattractant protein 1
*MDA*: Malondialdehyde
*MFG-E8*: Milk fat globule-EGF factor 8
*MIF*: Macrophage migration inhibitory factor
*MPO*: Myeloperoxidase
*MSA*: Mouse serum albumin
*MTTP*: Microsomal triglyceride transfer protein
*MUFA*: Monounsaturated fatty acids
*MMP9*: Matrix metalloproteinase- 9
*NCOA4*: Nuclear receptor coactivator 4
*NEK7*: NIMA-related kinase
*NET*: Neutrophil extracellular trap
*NLRP3*: Nod-like receptor protein 3
*NOX*: NADPH oxidase
*NK*: Natural killer
*Nrf2*: Nuclear factor erythroid 2-related factor 2
*OART*: Oxaliplatin-Artesunate
*OxPAPC*: Oxidized 1-palmitoyl- 2-arachidonyl-phosphatidylcholine
*Ox-LDL*: Oxidized low-density lipoprotein
*OxPC*: Oxidized phosphatidylcholine
*OxPE*: Oxidized phosphatidylethanolamine
*OxPS*: Oxidized phosphatidylserine
*PACM-βCD*: β-Cyclodextrin-polyacryloylmorpholine (PACM-βCD)
*PE*: Phosphatidylethanolamine
*PGE2*: Prostaglandin E2
*Pin1*: Peptidylprolyl cis/trans-isomerase A1
*pMHC I*: Peptide–MHC class I
*PMN-MDSC*: Polymorphonuclear myeloid-derived suppressor cell
*PUFA*: Polyunsaturated fatty acid
*RNS*: Reactive nitrogen species
*ROS*: Reactive oxygen species
*SAPE-OOH*: 1-Steaoryl- 2–15-HpETE-sn-glycero- 3-phosphatidylethanolamine
*SIRT*: Sirtuins
*SOCS*: Suppressor of cytokine signaling
*SREBP*: Steroid regulatory element-binding protein
*SSZ*: Sulfasalazine
*STAT*: Signal transducer and activator of transcription
*TGF-β*: Transforming growth factor β
*TIN*: Tumor-infiltrating neutrophils
*TLR*: Toll-like receptor
*TME*: Tumor microenvironment
*TNBC*: Triple-negative breast cancer
*Treg*: T regulatory cell
*TNF*: Tumor necrosis factor
*Tt-DDE*: 14 - 3- 3ζ, Trans- 2,4-decadienal
*VLCFA*: Very-long-chain fatty acids
*VKH2*: Vitamin K hydroquinone
*VKORC1L1*: Vitamin K epoxide reductase complex subunit 1 like 1
*XBP1*: X-box binding protein 1
*ZVI-NP*: Zero-valent iron nanoparticles
